# Supplementary material for: Ratiometric Fluorescent Sensor Based on Tb(III) Functionalized Metal-Organic Framework for Formic Acid
Source: Molecules. 2022 Dec 8;27(24):8702. doi: 10.3390/molecules27248702 (PMC9781586; doi:10.3390/molecules27248702)
Supplement: Supplementary file 1 [file molecules-27-08702-s001.zip › molecules-2065739-supplementary.pdf]

Ratiometric Fluorescent Sensor Based on Tb(III) Functionalized Metal-Organic Framework for Formic Acid

Chao-Wei Zhang †, Mei-Ling Li †, Yi-Duo Chen, Qi Zhou and Wei-Ting Yang *

Key Laboratory of Advanced Materials of Tropical Island Resources, Ministry of Education, School of Chemical Engineering and Technology, Hainan University, Haikou 570228, China
* Correspondence: yangwt@hainanu.edu.cn
† These authors contributed equally to this work.

S1. Materials and Methods

All the reagents and solvents were purchased from commercial sources and used directly without further purification. Infrared (IR) spectra were acquired with KBr discs in the range of 4000 to 400 cm^{-1} on Bruker TENSOR-27 IR spectrometer. Powder X-ray diffraction patterns were collected on Rigaku D/MAX X-ray powder diffractometer (Cu-Kα, 1.5418 Å) at room temperature. The solid state luminescent spectra were collected on a GangDong F-320 fluorescence spectrophotometer at room temperature. XPS measurements were performed in a stainless-steel ultrahigh vacuum chamber (PerkinElmer, model 5500, base pressure <10^{-10} mbar).

S2. LOD Calculations:

According to the equation of LOD = 3δ/k, the LOD of TH25 for FA is 2.1 ppm. The specific data are shown in Table S1.

Table S1. Fluorescence intensity data of TH25 measured at 414 nm, 545 nm for ten repeated measurements.

	I_{414}	I_{545}	I_{414}/I_{545}
1	63384.1	363191.2	0.1745199
2	61903.1	351728.1	0.175997
3	63762.7	366568.3	0.1739449
4	63443.5	365718.4	0.1734764
5	62886.3	360330.9	0.1745238
6	63131.4	362346.5	0.1742294
7	62510.1	361039.5	0.1731392
8	62898.4	361702.3	0.1738955
9	62462.4	359903.8	0.173553
10	62199.3	357084.3	0.1741866

Average (I_{414}/I_{545}) =1/10* (0.1745199+0.175997+0.1739449+0.1734764+0.1745238+0.1742294+ 0.1731392+0.1738955+0.173553+0.1741866) =0.174

δ= $[1/10^* \sum_1^{10} (I_{414}/I_{545}\text{-Average } (I_{414}/I_{545}))^2]^{1/2}$= 0.000751

S3. Supplementary characterizations

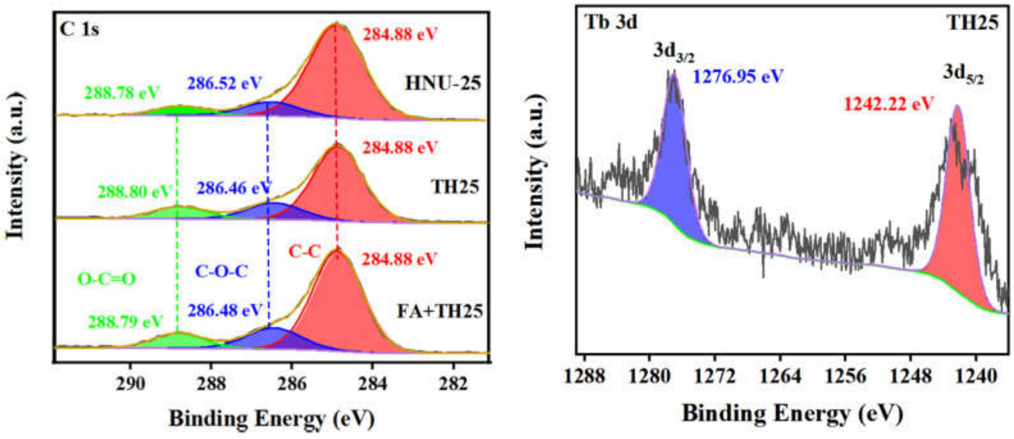

(**a**) (**b**)

Figure S1. High-resolution XPS spectra of C ls of HNU-25, TH25 and TH25+FA; (**b**) high-resolution XPS spectra of Tb 3d of TH25.

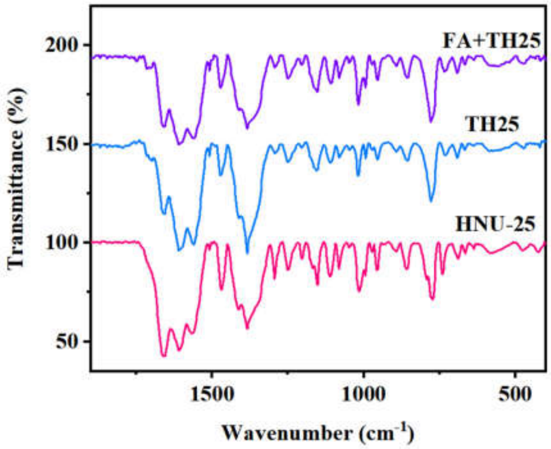

Figure S2. IR spectra of HNU-25, TH25 and FA+TH25.
